# Walls That Grow: Fungal Species-Driven Performance of Mycelium-Based Composites Grown on Rice-Husk Waste

**DOI:** 10.3390/polym18080932

**Published:** 2026-04-10

**Authors:** Zahra Parhizi, Ewa Jadwiszczak, John Dearnaley, Deirdre Mikkelsen, Paulomi (Polly) Burey

**Affiliations:** 1 Centre for Future Materials (CFM), University of Southern Queensland, Toowoomba, QLD 4350, Australia; zahra.parhizi@unisq.edu.au; 2 School of Science, Engineering and Digital Technologies, University of Southern Queensland, Toowoomba, QLD 4350, Australia; ewa.jadwiszczak@unisq.edu.au (E.J.); john.dearnaley@unisq.edu.au (J.D.); 3 School of Agriculture and Food Sustainability, University of Queensland, St. Lucia, QLD 4072, Australia; d.mikkelsen@uq.edu.au

**Keywords:** mycelium, rice husk, mechanical properties, fungal composite, biomaterial

## Abstract

Mycelium-based composites (MBCs) are gaining attention as sustainable alternatives to conventional materials because they are grown biologically rather than produced through resource-intensive extraction and processing. This study evaluates MBCs for non-load-bearing wall panels and environmentally responsible substitutes for traditional building materials. A reproducible manufacturing process is presented, and heat-pressed panels are characterised for physical, mechanical, and chemical performance. Novelty lies in species-driven evaluation using rice-husk waste as the sole lignocellulosic substrate and a Queensland-native *Amauroderma* species. Five fungal species, *Trametes hirsuta*, *Ganoderma* sp., *Amauroderma* sp., *Pycnoporus coccineus* and *Trametes versicolor*, were cultivated on rice husks and compared under identical processing conditions. Statistical analysis showed species selection significantly influenced tensile strength, whereas flexural and compressive performance showed no significant interspecies differences. Panels achieved tensile, compressive, and flexural strengths up to approximately 0.47, 0.35, and 1.35 MPa, respectively, with *Amauroderma* exhibiting the highest stiffness and compressive performance. Composites from four of the five species showed low moisture sensitivity and favourable thermal behaviour relative to previously reported mycelium materials. These results demonstrate that fungal species selection is a key design lever and supports rice-husk-derived MBCs as sustainable insulation and non-load-bearing construction materials.

## 1. Introduction

The engineering and construction sector is one of the most resource and energy-intensive industries worldwide [1]. It consumes nearly 50% of extracted mineral resources, is the largest contributor to landfill waste, and in 2024–2025 accounted for approximately 32% of global energy demand and 34% of anthropogenic CO_2_ emissions [2,3]. Traditional construction materials energy intensive production process involving the extraction of raw minerals contributes to resource depletion and imposes notable sustainability challenges, presenting long-term environmental issues for future generations [4,5]. Wood-based building materials such as plywood also exhibit substantial fossil-based global warming potential (GWP), primarily arising during the raw material supply phase [6].

Global population growth and accelerating urbanisation are driving a dramatic increase in waste generation. Annual waste production is projected to rise by 70%, from 2.01 billion tons in 2016 to approximately 2.20 billion tons by 2025 and 3.40 billion tons by 2050 [2]. With the steady rise in agricultural production, the volume of associated by-products, including rice husks, cotton stalks, and various crop straws, continues to increase. Despite their renewable nature, most of these lignocellulosic residues are still treated as waste and commonly disposed of or burned in the field, increasing the carbon footprint and additional gases that exacerbate the climate crisis [7,8].

Mitigating these impacts requires a transition from the prevailing linear production model (extract–manufacture–use–discard) to circular strategies that extend material lifespans and reduce environmental burdens [9]. Within this context, bio-based construction materials represent a promising class of sustainable alternatives. These materials can be derived from agricultural residues and other organic waste streams and are designed for recyclability or compostability eliminating the need for virgin resource extraction [5].

Bio-composites, which typically comprise natural fibres and bio-based binders, offer one such pathway [10]. A notable example is mycelium-based composites (MBCs), which are produced by inoculating lignocellulosic substrates with fungal mycelium. A broad range of applications is currently being explored for these biobased materials, including packaging [11], design tooling [12], furniture [13], paper products [14], building materials, textile films [15], insulation systems [16,17], sound-absorbing and flooring composites [18].

MBCs are produced by inoculating organic substrates with a fungal strain [19,20]. The vegetative mycelium colonises and degrades the substrate, metabolising the breakdown products to extend its hyphae [21]. This simultaneously forms an interwoven chitinous network that binds the particles. To support this growth, substrates must provide key nutrients along with sufficient water, which is the most critical factor [20,22]. Substrates are often derived from agricultural residues, wood by-products, or natural fibres such as flax and cotton [23].

Successful fabrication requires strict sterilisation of both the substrate and the growth environment to prevent contamination. After 2–3 weeks, the growth cycle is complete. At this stage, the material may be heat-treated (≥60 °C) to terminate growth or, alternatively, stored under ambient conditions to maintain the mycelium in a dormant state for potential reactivation [23]. Once complete colonisation is achieved, the fungal biomass is inactivated by thermal treatment, which also removes residual moisture, leaving behind a stable, lightweight, and biodegradable composite [5].

The structural anatomy of the mycelium, which differs across fungal species, has a critical impact on the mechanical performance of the composite. In basidiomycetes, hyphal systems can be monomitic (only generative hyphae), dimitic (generative and skeletal hyphae), or trimitic (generative, skeletal, and binding hyphae) [14]. It also has been reported that composites derived from trimitic fungi, such as *Trametes versicolor*, exhibit superior compressive, tensile, and flexural strength compared to those produced from monomitic fungi, such as *Pleurotus ostreatus* [24,25].

Among the key determinants of MBCs’ mechanical properties, fungal strains and substrate composition have consistently been identified as the dominant factors. Variability in these factors leads to considerable heterogeneity in reported mechanical strength results [26].

Despite growing interest in MBCs, two critical knowledge gaps continue to limit their translation into reliable, construction-oriented materials. First, the influence of fungal species selection on panel performance is not yet systematically defined, and robust species-resolved structure–property relationships remain scarce, hindering rational strain selection for targeted mechanical and functional outcomes. Second, rice husk, despite being a locally abundant agricultural waste stream, remains underutilised as an MBC feedstock, and the ways in which different fungal species interact with rice husk to govern microstructure development, interfacial bonding, and resulting mechanical, thermal, and moisture-related behaviour are largely unknown. In addition, the heavy reliance on commercial strains and pre-formulated substrates restricts reproducibility, limits mechanistic insight, and reduces regional applicability and scalability.

In this study, MBC panels were fabricated through a reproducible process by cultivating five locally sourced fungal species, *Trametes hirsuta*, *Ganoderma* sp., *Pycnoporus coccineus*, *Trametes versicolor*, and a Queensland-native *Amauroderma* sp., using rice husk waste as the sole growth substrate. The resulting composites were systematically evaluated through complementary microstructural, spectroscopic, thermal, wettability, and mechanical characterisation to quantify performance and establish fungal species as a primary design variable governing MBC behaviour. Both the fungal isolates and the rice husk feedstock were locally sourced and cultivated, demonstrating a practical route toward regionally scalable and circular composite production. In addition to performance benchmarking, biological safety was assessed and the potential for fungal regrowth under ambient humidity conditions relevant to construction service environments was evaluated to address a key barrier to real-world building deployment.

This study also advances a practical densification strategy by converting fully colonised biomass matrix into panel-grade materials via controlled heat-pressing, providing a reproducible route to reduce porosity and improve cohesion using widely accessible processing conditions. A defined rice-husk and wheat nutrient system was employed to support consistent colonisation prior to densification, enabling a scalable growth-to-panel manufacturing framework. Collectively, this work provides the first systematic comparison of five fungal species, including a locally isolated native Queensland *Amauroderma* strain, grown exclusively on rice husk and processed into heat-pressed panels. By linking microstructure and chemistry to mechanical behaviour, thermal degradation, and surface water repellence, the study delivers a comprehensive species-resolved performance map for rice-husk-derived mycelium composites directly informing the design of non-load-bearing wall panels, interior partitions and insulation boards. 

## 2. Materials and Methods

For this study, material combinations were chosen based on their availability, reliable supply, and compatibility with laboratory-scale processing. The selected fungal isolates and substrate represent practical and readily accessible options that support reproducible MBC manufacture while offering meaningful insight into the wider applicability and performance potential of these bio-derived composites.

### 2.1. Fungal Species and Cultures

In this study, overall, five different fungal species were used. *Trametes hirsuta*, *Pycnoporus coccineus* and *Amauroderma* sp. fruiting bodies were collected from Ravensbourne, Southeast Queensland, Australia and the University of Southern Queensland, Toowoomba campus, Queensland, Australia. Small pieces of fruiting bodies were sterilised with 70% ethanol to remove any contamination, then isolated on methyl 1-(butylcarbamoyl)-2-benzimidazolecarbamate, benomyl (Merck KGaA, Darmstadt, Germany) media as per the Worrall method [27]. All strains were kept in a Shell Lab incubator in 25 °C, humid and dark environment to grow. After initial isolation, a 1 cm^3^ cube of each colony was cut and transferred to Potato Dextrose Agar Thermo Scientific™ Oxoid™ PDA petri dishes (Thermo Fisher Scientific, Waltham, MA, USA) and incubated for 7 days to grow pure cultures.

In addition, two other cultures, *Trametes versicolor* and *Ganoderma*, were obtained from the University of Queensland fungal culture collection. The pure cultures were stored in ambient conditions and were sub-cultured onto PDA plates to initiate fresh growth.

To confirm that cultures met the criteria for subsequent composite fabrication, plates were assessed for absence of visible contamination, consistent colony morphology indicative of a pure culture after subculturing, and vigorous growth and uniform colonisation of the agar surface. Only cultures meeting these criteria were used to prepare grain spawn and inoculate substrates for panel production.

### 2.2. Substrate Preparation and Inoculation

The substrate was prepared using rice husk (CopRice©, Leeton, Australia) supplemented with non-fungicide wheat grains (PB Agrifood©, Toowoomba, Australia) as a nitrogen-rich additive, mixed at a volumetric ratio of 3:1. Rice husks and wheat grains were sterilised separately in 1 L Schott bottles containing 150 mL and 350 mL of Milli-Q water (Merck Millipore, Burlington, MA, USA), respectively, using a Tomy SX-500U Lab Autoclave (Tomy Lab, Tokyo, Japan) at 120 °C for 20 min. After sterilisation, the materials were drained and allowed to cool at room temperature for approximately 3 h.

For inoculation, ten 1 cm^3^ agar plugs of the fungal pure culture were introduced into 250 mL of sterilised wheat grains in a sealed 1 L Schott bottle, which was then incubated at 25 °C for 10 days to promote mycelial colonisation. During this period, the bottle was gently shaken periodically to improve aeration and facilitate uniform mycelial development.

Following the colonisation phase, 750 mL of sterilised rice husks were transferred into a 20 × 20 × 5 cm plastic clinical instrument tray serving as the mould and thoroughly mixed with the myceliated wheat grains to ensure even distribution throughout the substrate. The mould was then covered with aluminium foil and incubated at 25 °C for 14 days to allow complete colonisation and substrate digestion.

**Figure 1:**
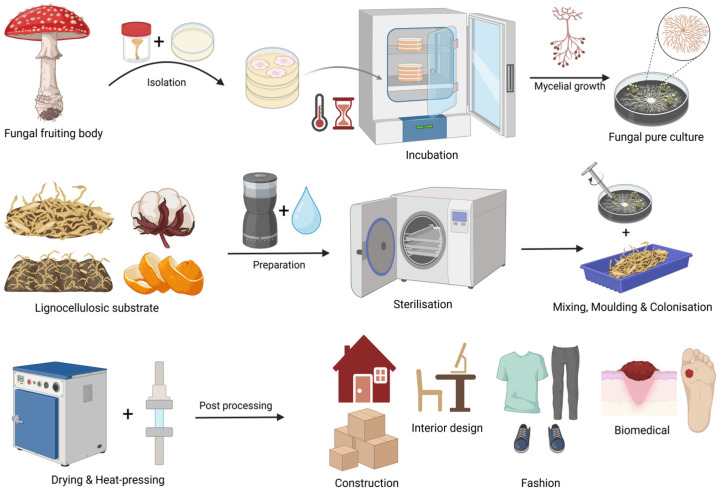
Fabrication process and applications of mycelium-based composites. Created in BioRender. Parhizi, Z. (2026) https://BioRender.com/gz36giu, accessed on 3 April 2026.

Figure 1 provides an overview of the fabrication process employed for producing MBCs in this study.

### 2.3. Material Fabrication

After two weeks of incubation, the harvested biomass was oven-dried at 60 °C for 72–96 h, with the duration adjusted according to fungal species and residual moisture content. This drying step effectively terminated fungal activity, rendered biomass inert, and ensured its suitability for subsequent post-processing stages. The dried biomass was then transferred from the clinical instrument trays into a 20 × 20 cm aluminium pressing mould and hot-pressed at 120 °C under 3 tonnes of pressure for 15 min using a CARVER Hydraulic Unit 3925 (Carver, Inc., Wabash, IN, USA) to produce consolidated flat composite panels. To assess the biological safety of the fabricated materials and evaluate the potential for fungal regrowth under ambient humidity conditions relevant to construction applications, all heat-pressed MBC panels produced from the five fungal species were placed on PDA plates under both sealed and unsealed conditions and incubated in darkness at controlled relative humidity for 20 months. No fungal regrowth was observed, confirming that the heat-pressed composites were biologically inert and safe for use as construction materials.

### 2.4. Sample Preparation

Specimens were prepared using an electric rotary shear blade and cut into dog-bone geometries for tensile testing, rectangular samples for flexural and compressive testing, 1 × 1 cm sections for microscopic imaging, and small fragments (5–10 mg) for morphological and compositional analyses. Fragments were taken from the heat-pressed panel bulk; for SEM imaging, samples were ground to expose a representative internal fracture-like surface prior to mounting, while for FTIR analysis, material was ground into a fine homogeneous powder to reduce compositional heterogeneity. Accordingly, the micrographs presented in this study primarily reflect the internal microstructure of the heat-pressed panels rather than the as-pressed external surface. All samples were measured, cut, and tested in accordance with ASTM D1037 [28], the most applicable standard for evaluating the mechanical performance of wood- and lignocellulosic-based composite materials.

**Figure 2:**
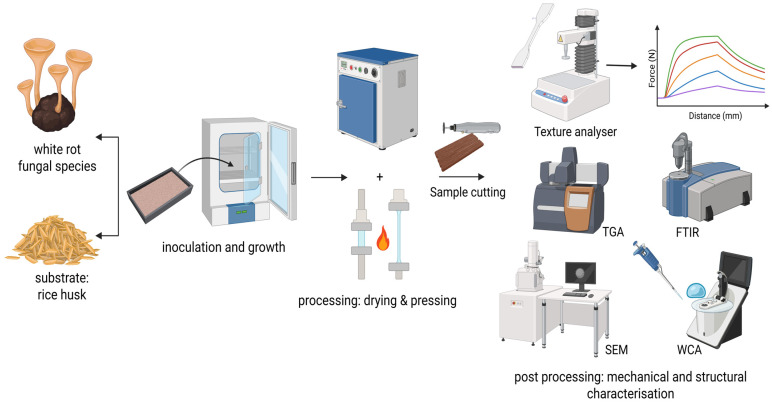
Summary of the analytical techniques used to characterise the MBCs produced in this study. Created in BioRender. Parhizi, Z. (2026) https://BioRender.com/w0jqycu, accessed on 3 April 2026.

Prior to detailing the testing programme, Figure 2 provides an overview of all analytical procedures applied to the MBCs in this study, including physical characterisation, mechanical testing, thermal analysis, and chemical and structural examination.

### 2.5. Morphological and Microstructural Analysis

Morphological characteristics of the specimens were examined using scanning electron microscopy (SEM) JEOL JCM-7000 (JEOL, Tokyo, Japan). Prior to imaging, samples were sputter-coated with a thin gold layer for 1 min. Each specimen was cut into 6 × 6 mm sections and placed onto a 10 mm diameter aluminium holder using double-sided conductive adhesive. Surface topography and microstructural characteristics were further investigated using a high-resolution optical microscope (Olympus DSX1000, Olympus, Tokyo, Japan).

### 2.6. Chemical Characterisation

Infrared spectra were collected using a ThermoScientific Nicolet™ iS50 FT-IR Fourier Transform Infrared (FTIR) spectrometer (Thermo Fisher Scientific, Waltham, MA, USA) equipped with a single-reflection diamond attenuated total reflection (ATR) accessory. For each sample, spectra were obtained to characterise the dominant chemical functional groups, molecular interactions, and contributions from the biomass constituents. All spectra were recorded over the accessible mid-infrared region (4000–1500 cm^−1^).

### 2.7. Thermal Degradation Analysis

The thermal decomposition behaviour of the composites was evaluated using thermogravimetric analysis (TGA) on a TA Instruments SDT 650 (New Castle, DE, USA). Approximately 5–10 mg of each specimen was placed in a 90 µL high-temperature alumina crucible and heated from 25 to 600 °C at a constant rate of 30 °C min^−1^ under a nitrogen purge (50 mL min^−1^).

### 2.8. Physical and Mechanical Analysis

Specimens were cut to the required dimensions following ASTM D1037 [28] using an electric rotary shear cutter (DREMEL, Mount Prospect, IL, USA). Tensile, compressive, and flexural tests were performed using a TA.XTplus100C Texture Analyser (Stable Micro Systems, Godalming, UK) with the appropriate fixtures and grips for each testing configuration. Across all mechanical tests, slight irregularities in the contact interfaces were unavoidable due to the inherently rough surfaces of the MBC panels. Owing to culture growth limitations encountered during this study, *Trametes versicolor*-based panels could not be fabricated for mechanical testing; therefore, analyses were conducted using MBC panels produced from the remaining four fungal species.

#### 2.8.1. Tensile Strength

For each species-derived panel, three replicate specimens were tested. For performing tensile tests, the Miniature Tensile Grip was used, and the samples were machined into a standard dog-bone shape with a parallel width of 51 mm and an overall length of 254 mm. The thickness of each specimen was measured using a KINCROME digital calliper. Tests were performed under a maximum load capacity of 1 kN at a crosshead speed of 3 mm/min. The recorded load–displacement data were subsequently converted into stress–strain curves using the following equations [28]:

(1) $$\sigma =\frac{F}{A}$$

and

(2) $$\varepsilon =\frac{\Delta L}{L_{{}^{\circ}}}$$

where σ is the tensile stress (MPa), ε is strain, F is the applied force (N), A is the initial cross-sectional area (mm^2^), ΔL is the elongation between the loading surfaces (mm), and $L_{{}^{\circ}}$ is the original gauge length of the specimen (mm).

#### 2.8.2. Compressive Strength

Compression testing was conducted using a bench-top texture analyser equipped with a 1 kN load cell to ensure measurement accuracy. A constant displacement rate of 0.5 mm/min was applied. Testing stopped once the specimen reached a predetermined strain level between 75 and 85%. The modulus of elasticity in compression tests was calculated as

(3) $$E_{c}=\frac{L_{{}^{\circ}}}{bd}\frac{\Delta F}{\Delta L}$$

where $E_{c}$ is the modulus of elasticity (Young’s modulus) measured in Pa, ΔF is the change in applied force (N), ΔL is the change in deformation (elongation), b is the width of the specimen cross-section (mm), d is the thickness of the specimen cross-section (mm), and $L_{{}^{\circ}}$ is the original gauge length of the specimen (mm).

Stress–strain curves were derived from the load–displacement data using the following relationships:

(4) $$\sigma =\frac{F}{A}$$

and

(5) $$\varepsilon =\frac{\Delta L}{L_{{}^{\circ}}}$$

where F denotes the compressive force (N), ε is strain, A is the initial cross-sectional area of the specimen (mm^2^), ΔL is the displacement between loading surfaces (mm), and $L_{{}^{\circ}}$ is the original height of the specimen (mm).

#### 2.8.3. Flexural Strength

Flexural behaviour was assessed using a three-point bending configuration on the same test machine, operating at a crosshead speed of 3 mm/min. This setup subjected the specimen to tensile loading on the bottom surface and compressive loading on the top surface, enabling a comprehensive assessment of the material’s stiffness. The span length was 100 mm, calculated according to the nominal thickness of each panel and as the limiting span of the available equipment. Because the specimen thickness varied slightly among samples, the applied loading rate also differed accordingly. For reference, the average loading rate of flexural specimens was 3 mm/min. Each test was terminated upon specimen failure or when the applied load decreased to below 20% of the maximum recorded value. The modulus of rupture, MOR ($F_{m}$) was calculated using

(6) $$F_{m}=\frac{3P_{max}l_{1}}{2bd^{2}}$$

where $P_{max}$ is the maximum load (MPa), $l_{1}$ is the support span (mm), b the specimen width (mm) and d is the specimen thickness (mm). Apparent elastic modulus, MOE (E) was calculated using:

(7) $$E=\frac{L^{3}}{4bd^{3}}\frac{\Delta P}{\Delta y}$$

where L is the length of the span (mm), b the specimen width (mm) and d is the specimen thickness (mm) and $\frac{∆P}{∆y}$ represents the slope of the linear section of the stress–strain curve.

#### 2.8.4. Density

Density was determined using a gas pycnometer (AccuPyc III 1350, Micromeritics, Norcross, GA, USA). Each specimen was measured over five cycles, and the mean value was reported as the final density. Composites were cut into small fragments to occupy ~80% of the 3.5 cm^3^ sample cup, which was then sealed with a cap and placed into the analysis chamber. Nitrogen was used as the operating gas, and measurements were conducted at an operating pressure of 19.5 psig.

### 2.9. Wettability Performance

The wettability of the samples was evaluated by static water contact angle (WCA) measurements using the sessile drop method. Measurements were performed using a high-resolution optical microscope (Olympus DSX1000). To assess surface hydrophobicity and water interaction behaviour of the samples, a 1 µL droplet of Milli-Q water was carefully deposited onto the surface of each specimen, including fungal biomass prior to heat pressing and the final panels after heat pressing, using a micropipette. The droplet profile was captured 5 s after deposition and analysed using ImageJ^®^ software (version 1.54g) to determine the contact angle. For each specimen, at least three measurements were recorded to ensure measurement reliability and reproducibility.

### 2.10. Statistical Analysis

Statistical analyses were performed to evaluate species-dependent differences in the mechanical performance of the mycelium-based composites. All results are reported as mean ± standard deviation, calculated from three independent replicates per fungal species. Descriptive statistics were used to summarise flexural, tensile, and compressive strength data.

A one-way analysis of variance (ANOVA) was conducted to assess whether statistically significant differences existed among fungal species for each mechanical property. The null hypothesis assumed no significant difference between group means. Statistical significance was defined at a significance level of α = 0.05. Where the ANOVA indicated a significant effect (*p* < 0.05), post hoc pairwise comparisons were performed using Tukey’s honestly significant difference (HSD) test to identify specific group differences.

All statistical analyses were conducted using the Data Analysis Toolpak in Microsoft Excel (Microsoft Corporation, Redmond, WA, USA). Assumptions of ANOVA, including independence of observations, continuity of dependent variables, and homogeneity of variances, were assessed prior to analysis. Given the inherent variability of non-homogeneous bio-based materials and the limited sample size, results are interpreted with appropriate caution.

## 3. Results and Discussion

### 3.1. Morphological and Structural Analysis

**Figure 3:**
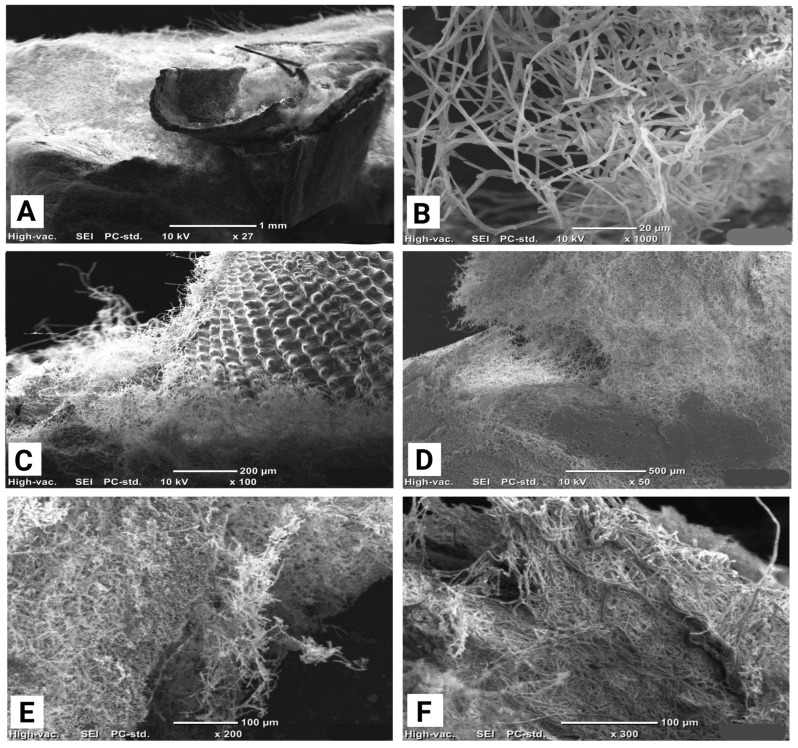
Scanning Electron Microscope image of MBCs made by (**A**) *Trametes hirsuta*, (**B**) *Ganoderma* sp., (**C**,**D**) *Pycnoporus* sp., (**E**) *Amauroderma* and (**F**) *Trametes versicolor*.

SEM micrographs of MBCs produced using different fungal species are presented in Figure 3A–F across multiple magnification levels. As expected for a naturally derived and inherently non-homogeneous material, mycelium distribution varied across each sample.

At low magnification (Figure 3A), a continuous mycelial layer is visible on the *Trametes hirsuta* panel surface, bridging adjacent lignocellulosic particles and indicating effective interfacial cohesion. The higher magnification cross-sectional image of the *Ganoderma*-based panel in Figure 3B further confirms internal colonisation, where an interconnected hyphal network permeates the bulk matrix [5]. Such internal colonisation can promote more distributed load transfer and progressive damage development by increasing the number of particle–hyphae contact points and frictional interlocking pathways during deformation. In the *Pycnoporus* samples (Figure 3C,D), hyphae are observed firmly adhering to wheat grain debris, demonstrating strong nutrient–substrate–fungus interactions; notably, Figure 3D also shows substantial mycelial accumulation extending deep into the composite, indicative of thorough bulk colonisation rather than surface-limited growth. Microstructural heterogeneity is evident in the *Amauroderma* sample (Figure 3E), where loosely organised hyphal bundles transition into more compact and densely interwoven regions, consistent with natural variations in substrate porosity and particle arrangement. Regions of densely interwoven hyphal bundles are expected to improve stiffness and peak strength by increasing the effective load-bearing network density and reducing interfacial slip. At the highest magnification (Figure 3F), multilayered hyphal networks in the *Trametes versicolor* panel form rope-like aggregates that wrap around individual substrate particles, contributing to enhanced mechanical interlocking within the matrix [29]. These SEM observations confirm extensive fungal colonisation throughout both the surface and interior of the MBCs, with species-dependent differences in hyphal density, spatial distribution, and network architecture reflecting distinct fungal growth behaviours and developmental dynamics [25].

Collectively, these microstructural differences provide a mechanistic basis for the species-dependent tensile stiffness, compressive load-bearing capacity, and post-peak responses reported in the mechanical results.

**Figure 4:**
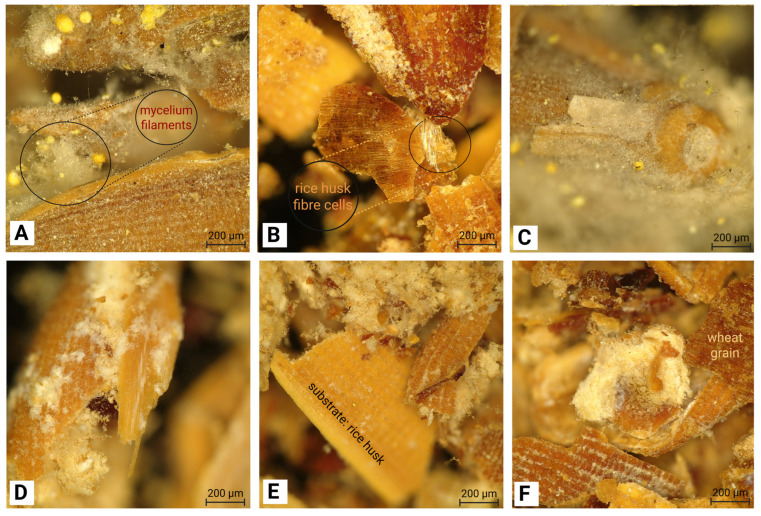
Microstructural characteristics of MBCs produced by (**A**) *Amauroderma*, (**B**) *Ganoderma* sp., (**C**) *Pycnoporus* sp., (**D**,**E**) *Trametes hirsuta*, and (**F**) *Trametes versicolor* using a high-resolution optical microscope. Scale bar = 200 μm.

To further investigate the surface topography microstructural characteristics of MBC panels, high-resolution optical microscopy images (Figure 4A–F) highlight the non-homogeneous yet functionally integrated nature of mycelial development across different fungal species.

In the *Amauroderma* panel (Figure 4A), early colonisation is evident through fine hyphal networks bridging rice husk fragments accompanied by small, yellow-pigmented structures indicative of active growth. The *Ganoderma* sample (Figure 4B) shows progressive colonisation, where hyphae exhibit moderate attachment and penetration along fractured substrate edges. Dense, cotton-like mycelial masses encapsulating rounded or tubular substrate particles are observed in the *Pycnoporus* panel (Figure 4C), suggesting strong localised adhesion [5]. In the *Trametes hirsuta* samples (Figure 4D,E), uniformly distributed hyphae bind tightly to exposed fibrous layers, while adjacent regions display heterogeneous colonisation with patches of dense growth next to minimally colonised surfaces, reflecting infiltration of the mycelium into the substrate core. The *Trametes versicolor* panel (Figure 4F) demonstrates the most advanced colonisation, where thick mycelial clusters surround substrate particles, effectively filling void spaces and contributing to composite consolidation [30].

**Figure 5:**
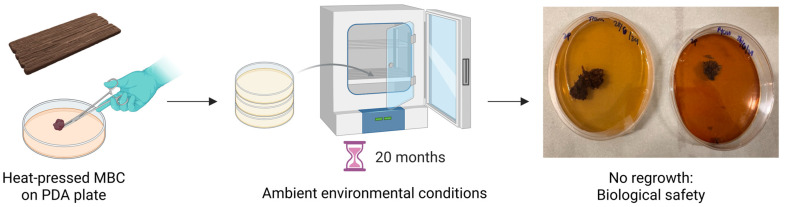
Biological stability assessment for heat-pressed MBC panels. Created in BioRender. Parhizi, Z. (2026) https://BioRender.com/g0dwweo, accessed on 3 April 2026.

Biological safety and stability were evaluated as shown in Figure 5 by placing heat-pressed MBC panels produced from all five fungal species onto PDA plates under both sealed and unsealed conditions, followed by incubation in darkness at controlled relative humidity for 20 months. No fungal regrowth was observed throughout the monitoring period, indicating that the heat-pressed panels remained inert and biologically stable with respect to fungal reactivation under the conditions assessed.

### 3.2. Chemical Characterisation

**Figure 6:**
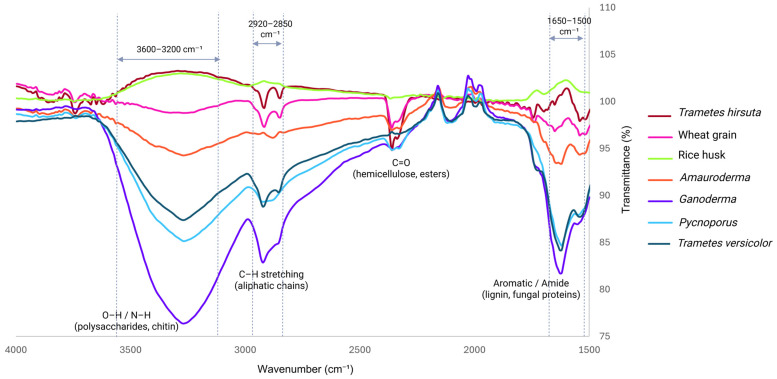
FTIR spectra of MBCs generated using different fungal species, with wheat grain and rice husk included for comparison as the nutrient source and primary substrate, respectively.

The FTIR spectra of MBC panels produced using different fungal species, together with those of the raw lignocellulosic substrate (rice husk) and the nitrogen-rich nutrient source (wheat grain), are shown in Figure 6. These spectra provide insight into the chemical composition of the resulting composites, the biochemical differences among fungal species, and their influence on lignocellulosic substrate degradation, which is governed by species-specific enzymatic activities and substrate utilisation patterns. The spectral region between 4000 and 1500 cm^−1^ highlights characteristic functional groups associated with both lignocellulosic components and fungal biomass.

Broad absorption bands observed in the 3300–3400 cm^−1^ region are attributed to O–H and N–H stretching vibrations. Variations in band intensity reflect differences in polysaccharide and protein content, as well as hydrogen-bonding interactions among hydroxyl groups [31,32]. Notably, *Ganoderma*, *Pycnoporus*, and *Trametes versicolor* exhibited the deepest transmittance minima in this region, indicating higher fungal biomass accumulation and more extensive hydrogen bonding. These spectral features are consistent with the dense mycelial networks observed in microscopic analyses. Changes in this region are also consistent with altered hydrogen-bonding environments at the mycelium–substrate interface, supporting physical interaction and interfacial adhesion contributions. However, these FTIR features do not directly confirm covalent crosslinking between fungal biomass and rice husk.

The C–H stretching vibrations at approximately 2920 and 2850 cm^−1^ were present in all samples and displayed similar spectral profiles, suggesting the conservation of aliphatic backbone structures associated with cellulose, hemicellulose, and fungal lipids across species. These bands are assigned to CH_2_ symmetric stretching and CH_2_OH groups in cellulose [33]. However, minor intensity differences indicate variations in substrate utilisation. *Ganoderma* and *Trametes versicolor* showed slightly enhanced aliphatic signals, which may reflect increased carbohydrate exposure resulting from partial delignification.

A distinct carbonyl (C=O) absorption near 1730 cm^−1^ was most pronounced in wheat grain, *Amauroderma*, and *Trametes hirsuta*. This band is commonly associated with acetyl groups in hemicellulose, xylan components, or oxidative modifications arising from fungal metabolic activity [34]. In contrast, the reduced C=O intensity observed in *Pycnoporus* and *Trametes versicolor* suggests greater hemicellulose consumption or deacetylation, consistent with their well-documented enzymatic capabilities.

The spectral region between 1600 and 1500 cm^−1^ corresponds to aromatic skeletal vibrations of lignin. In particular, C=C stretching of syringyl units appears between 1560 and 1520 cm^−1^, while the guaiacyl aromatic ring vibrations are observed at slightly lower wavenumbers [33,34,35]. Rice husk exhibited the strongest aromatic features, reflecting its higher lignin content. In contrast, fungal composites showed attenuated lignin-associated bands, with the greatest reductions observed for *Pycnoporus* and *Trametes versicolor*, indicating more effective lignin depolymerisation. Conversely, *Amauroderma* and *Ganoderma* retained more pronounced aromatic absorptions, suggesting lower lignin degradation or differences in selective decay mechanisms.

Overall findings of the FTIR analysis reveal that while fungal species induce measurable chemical modifications in the composites, the differences among species are relatively subtle. The inherent biological diversity of the fungi resulted in only minor variations in the overall chemical composition of the MBC panels. Because the absolute fungal biomass fraction was not directly quantified, the observed spectral differences may reflect a combination of species-dependent biomass accumulation and species-dependent substrate modification.

### 3.3. Thermal Degradation Analysis

**Figure 7:**
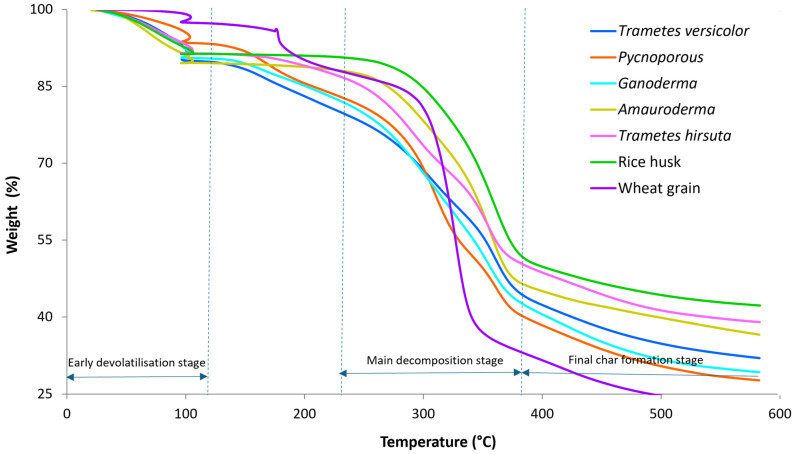
TGA mass loss curve for MBCs grown on different fungal species between 20 and 600 °C under nitrogen gas.

Results of the mass loss during thermal decomposition, measured by TGA, are shown in Figure 7. All MBCs produced with *Trametes versicolor*, *Pycnoporus*, *Trametes hirsuta*, *Amauroderma*, and *Ganoderma*, alongside the raw substrate components, rice husk and wheat grain, exhibit a multi-stage degradation profile characteristic of lignocellulosic biocomposites. This behaviour reflects the combined thermal responses of plant-derived constituents and fungal biomass. An initial minor mass loss occurs at temperatures below 150 °C and is attributed primarily to the evaporation of physically adsorbed moisture and the release of other low-volatility components. The most pronounced mass loss is observed in the mid-temperature range of approximately 250–380 °C, corresponding to the dominant thermal degradation of carbohydrate-rich fractions, including hemicellulose and cellulose, alongside the decomposition of fungal biopolymers [36]. At higher temperatures (380–600 °C), mass loss proceeds more gradually, consistent with the continued breakdown of thermally stable components and the formation of carbonaceous residues under inert conditions.

Although all composites display broadly similar degradation pathways, clear species-dependent differences are evident in the progression of the main decomposition stage and in the residual mass retained at 600 °C. In particular, the *Trametes versicolor* and *Pycnoporus* composites exhibit a greater overall mass loss by 600 °C, indicating a lower retained fraction at elevated temperatures [37]. In contrast, composites produced with *Amauroderma* and *Trametes hirsuta* retain a higher mass fraction in the upper temperature range, suggesting enhanced residue formation and a greater contribution from thermally persistent fractions following fungal growth and processing [25]. The higher char residue in *Amauroderma* and *T. hirsuta* panels indicates an increased char-forming tendency under the tested conditions, which is associated with improved thermal shielding during heat exposure. These results also support the potential suitability of these materials for low-carbon construction applications where passive fire performance is desirable [37]. The *Ganoderma* composite displays an intermediate response, closely aligning with the average behaviour observed across the MBC set.

The substrate controls exhibit distinct thermal behaviours that bracket those of the composites. Wheat grain shows a comparatively abrupt mass loss in the low-to-mid 300 °C range, consistent with a higher content of readily decomposable organic constituents under nitrogen. In comparison, rice husk retains a greater mass fraction at elevated temperatures and undergoes a more gradual degradation, reflecting increased thermal stability and higher char formation, likely associated with its silica-rich composition [25,37].

Overall, these results demonstrate that fungal species selection plays a significant role in governing the thermal stability and residue formation behaviour of MBCs. The observed differences in degradation temperature ranges and residual mass suggest that tailored fungal–substrate combinations may be exploited to optimise MBC performance for applications such as sustainable thermal insulation, where thermal resistance and char formation are desirable attributes [36,38].

### 3.4. Physical and Mechanical Analysis

#### 3.4.1. Tensile Strength

The four MBCs made from *Amauroderma*, *Ganoderma*, *Trametes hirsuta* and *Pycnoporus* showed different behaviour during the mechanical testing procedure (Table 1).

**Figure 8:**
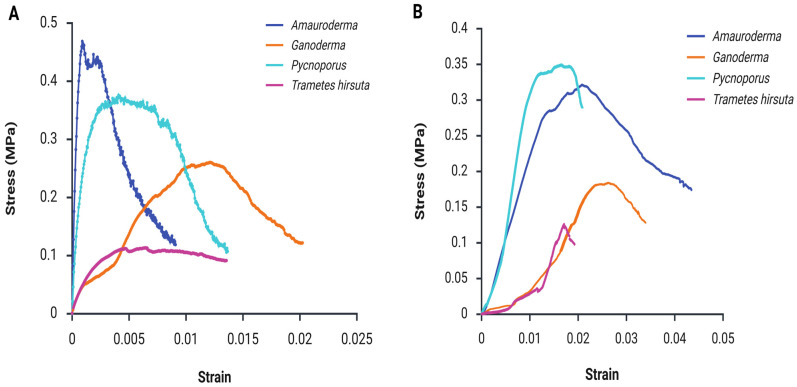
Tensile (**A**) and compressive (**B**) strength of MBCs made from different fungal species grown on rice husks.

The tensile stress–strain responses of the panels exhibited clear, species-dependent behaviour (Figure 8A). As shown in the figure, the *Amauroderma*-based panel demonstrated the steepest initial slope, corresponding to the highest stiffness, peak stress, and Young’s modulus among all samples (Table 1). Stress increased rapidly to an early maximum of approximately 0.47 MPa at a low rupture strain of 0.001, followed by pronounced post-peak softening, with stress gradually decreasing until a strain of 0.009. This response is characteristic of brittle tensile failure, suggesting rapid stress transfer and strong initial interfacial bonding within the composite [25]. This behaviour is consistent with the SEM observations of more densely interwoven hyphal bundles in the *Amauroderma* panel, which are expected to increase particle bridging density and stiffness.

The *Pycnoporus*-based panel exhibited a similar but less intense response, reaching an early peak stress of around 0.38 MPa at a low strain of 0.005, followed by a relatively stable post-peak region up to a strain of 0.012. This behaviour indicates intermediate tensile strength with a broader peak region but enhanced ductility compared to *Amauroderma* [39]. The broader post-peak region is consistent with the frictional pull-out of hyphal bridges observed in Figure 3C,D. In contrast, the *Ganoderma*-based panel demonstrated the most ductile behaviour, characterised by a gradual increase in stress over a wider strain range. The curve shows a sustained rise to a maximum stress of approximately 0.26 MPa at a strain of 0.012, followed by a slow and progressive decline to 0.12 MPa at higher strains (0.02). This response reflects stable load transfer, progressive damage mechanisms, and enhanced energy absorption capacity [9,29]. *Trametes hirsuta* exhibited the weakest tensile performance, with low stress levels with the maximum of 0.12 MPa, a limited strain capacity (0.014) and the lowest Young’s modulus (Table 1), as evident from the shallow slope and early plateau. This behaviour indicates inefficient stress transfer and early interfacial failure, leading to low tensile strength. Nevertheless, the broad peak and extended post-peak plateau demonstrate that this fungal species–based panel exhibited the highest ductility among the samples [36].

#### 3.4.2. Compressive Strength

The compressive stress–strain behaviour of the MBC panels was evaluated to assess their resistance under applied compressive loading (Figure 8B). Distinct, species-dependent responses were observed, indicating that fungal type strongly influences the composite load-bearing network, densification behaviour, and damage evolution mechanisms.

The *Pycnoporus*-based panel exhibited the highest compressive performance, characterised by a steep initial slope followed by progressive strain hardening and localised stress fluctuations. The stress increased to a peak of 0.35 MPa before entering a broad softening region at higher strains (Figure 8B). This response suggests efficient compressive load transfer, progressive densification of the microstructure, and stable load-bearing behaviour [31]. According to the micrographic analysis, strong bulk colonisation enables the porous network to collapse gradually under compression, increasing contact points during densification and stabilising the crushing response. Similarly, the *Amauroderma*-based panel demonstrated a comparable compressive response, with a gradual increase in stress leading to a wide plateau region at approximately 0.32 MPa. The extended plateau indicates pronounced ductility, improved damage tolerance, and high material toughness, reflecting a robust compressive load-bearing network. The superior compressive (Figure 8B) and tensile performance (Figure 8A) of the Amauroderma panels is consistent with SEM observations (Figure 3E), which show more densely interwoven hyphal bundles. Such microstructural features are expected to improve particle bridging and interfacial stress transfer, contributing to increased stiffness and higher peak stress under loading [30,31]. The *Ganoderma*-based panel exhibited a qualitatively similar response but with substantially lower compressive strength, reaching a peak stress of approximately 0.20 MPa, followed by a reduction in load-bearing capacity. This behaviour suggests limited structural reinforcement and inefficient stress transfer under compressive loading [30]. The *Trametes hirsuta*-based panel showed lower stress levels and a broad pre-peak region before reaching a maximum stress of approximately 0.13 MPa, followed by a relatively sharp post-peak decline. This gradual response indicates moderate damage tolerance with limited capacity for sustained compressive load bearing [25,40]. The comparatively weaker performance may be attributed to species-dependent differences in hyphal architecture and bonding density (Figure 3A), which likely reduce effective reinforcement and load transfer within the composite network. Compared with expanded polystyrene (EPS) foams, which typically exhibit compressive strengths in the range of 0.03–0.69 MPa [41], the MBC panels produced in this study showed compressive strengths of 0.13–0.38 MPa under the tested conditions, placing them within the lower-to-mid range of EPS grades while offering a bio-based alternative.

From an application perspective, particularly for non-load-bearing wall panel systems, these results indicate that *Amauroderma*-based MBCs exhibit superior ductility and post-peak load retention, suggesting greater damage tolerance and energy absorption capacity, while *Pycnoporus*-based MBCs provide the highest compressive strength and a stable crushing response [30].

#### 3.4.3. Flexural Strength

**Figure 9:**
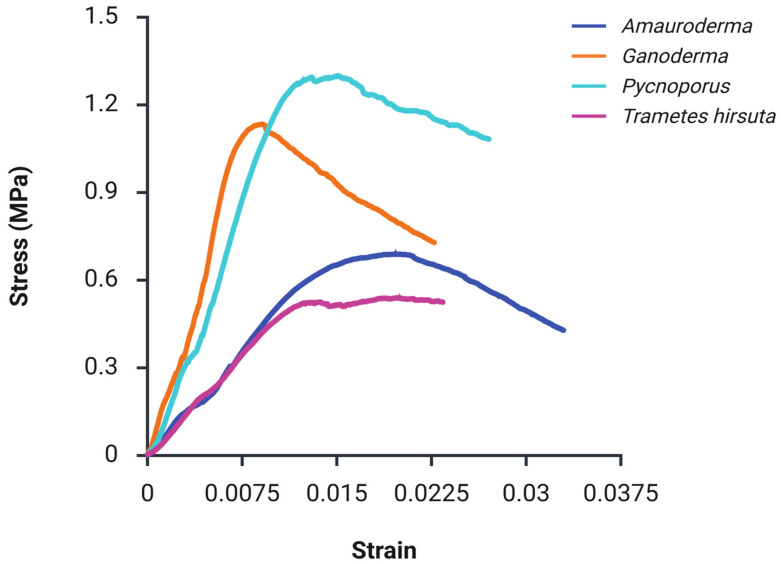
Flexural stress–strain behaviour of MBCs made from different fungal species.

Distinct trends in stiffness and maximum stress were observed during three-point bending compared with the tensile and compressive responses (Figure 9). The produced MBC panels exhibited flexural strengths ranging from approximately 0.6 to 1.38 MPa and flexural moduli between 172 and 532 MPa (Table 1), confirming species-dependent mechanical performance.

The *Pycnoporus*-based panel demonstrated the highest flexural strength, reaching a peak stress of 1.35 MPa, followed by a slow post-peak softening region, indicating stress redistribution under bending [42]. Correspondingly, this composite showed the highest MOE of 532.91 MPa, indicating superior stiffness and resistance to deformation. The *Ganoderma*-based panel also exhibited relatively high flexural strength with the greater MOE, peaking at 1.15 MPa. The stress–strain response shows a steeper post-peak decline, suggesting reduced flexural toughness compared to *Pycnoporus*. The *Amauroderma*-based panel displayed moderate flexural performance, with a maximum stress of 0.70 MPa and an extended strain capacity, indicating a more ductile bending response. In contrast, the *Trametes hirsuta*-based panel had the lowest flexural strength (˂0.6 MPa) and a relatively flat post-peak plateau, reflecting limited resistance to bending-induced damage.

From an application perspective, three-point bending is particularly relevant for non-load-bearing wall panels, as flexural loading represents the dominant stress mode in service [43,44]. Notably, all panels exhibited higher ultimate rupture strains under bending compared to tensile loading (Figure 8A and Figure 9), highlighting their improved deformation tolerance under flexural stresses. The flexural strength and modulus values obtained in this study fall within the range reported in the previous literature [45], and further confirm the intrinsic self-binding capability of mycelium in composite fabrication [42]. Moreover, compared with EPS boards and foams exhibiting flexural strengths in the range of 0.07–0.7 MPa [41], the MBCs produced in this study showed flexural strengths of 0.6–1.38 MPa under the tested conditions, overlapping the upper end of the EPS range and exceeding it for the highest-performing formulations while offering a bio-based alternative. While plywood commonly shows higher flexural strength (1.91–2.90 MPa) [46], this range approaches the lower end of plywood, supporting the use of these panels in non-load-bearing applications.

#### 3.4.4. Density

The extent of mycelial development and structural consolidation within the composite can be quantitatively assessed using density profile analysis. Optimal densification is influenced by several processing parameters, including particle size and distribution, curing duration, and fabrication conditions. These factors collectively govern the density and mechanical integrity of the material. Table 2 presents detailed experimental parameters, including average density values, sample masses, and specimen volumes, categorised by fungal species cultivated on wheat grain and rice-husk substrate.

The results clearly demonstrate that, even when grown on the same substrate, different fungal species yield MBCs with distinct density values, ranging from 250.137 to 259.140 kg/m^3^. The highest density was recorded for MBCs produced with *Amauroderma*, with MBCs fabricated from *Pycnoporus* exhibiting the lowest values. This trend is consistent with previous studies reporting a strong influence of fungal species on MBC density [12,47,48].

In addition to fungal strain, MBC density is affected by multiple factors, including substrate composition and particle size, growth conditions and duration, post-processing techniques, mould geometry, and drying protocols [40,49]. Despite inherent biological variability, the density of the MBC panels in this study lies within the range reported for lightweight polymer foams used in building applications (11–920 kg/m^3^) and is lower than typical plywood, which commonly exhibits densities of approximately 512–596 kg/m^3^ [14,50,51,52]. This suggests that MBCs have potential for application across a range of sectors, particularly in construction. Together, the density and Young’s modulus of the MBC panels fabricated from different fungal species in this study are consistent with the ranges reported for heat-pressed mycelium-based materials in the literature [25,53].

### 3.5. Wettability Performance

**Figure 10:**
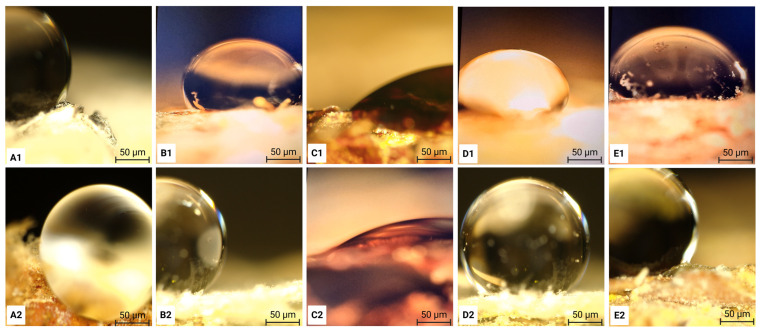
Wettability and water contact angle images of oven-dried biomass (**A1**–**E1**) cultivated by *Amauroderma*, *Ganoderma*, *Trametes versicolor*, *Trametes hirsuta*, and *Pycnoporus*, respectively, and the corresponding heat-pressed MBCs (**A2**–**E2**) produced from these fungal species. Scale bar = 50 µm.

The resulting WCA images are presented in Figure 10. The top row (A1–E1) shows the wettability behaviour of oven-dried (non-heat-pressed) fungal biomass cultivated by *Amauroderma*, *Ganoderma*, *Trametes versicolor*, *Trametes hirsuta*, and *Pycnoporus*, respectively, while the bottom row (A2–E2) displays the corresponding heat-pressed MBC panels. Water contact angle measurements were performed on a flat edge of a randomly selected cross-sectional area of each panel to assess surface hydrophobicity.

The oven-dried biomass samples exhibited less rounded droplet shapes, indicating partial water absorption into surface voids and reflecting the heterogeneous and highly porous nature of the fungal biomass. In contrast, after heat pressing, the composite surfaces became flatter and more compact, with reduced internal air voids. As a result, water droplets remained more spherical and showed minimal absorption, indicating increased surface hydrophobicity and a more consistent mycelium layer on the surface [30,54]. An exception was observed for the *Trametes versicolor*-based panel, which exhibited greater wettability compared to the other heat-pressed composites. These results demonstrate that heat pressing significantly alters the surface morphology and moisture interaction behaviour of MBCs by reducing porosity and limiting water penetration.

To evaluate the water-repellent behaviour of the manufactured MBC panels for potential construction applications, quantitative WCA results are summarised in Table 3. Contact angles above 90° indicate a more hydrophobic, water-repellent surface, whereas angles below 90° reflect increased wettability and greater affinity for water. As moisture sensitivity can adversely affect structural stability and durability, lower contact angles may indicate a higher risk of water uptake and reduced suitability for building-related use without additional surface treatment or protection [54].

### 3.6. Statistical Comparison of Fabricated MBCs

The statistical analysis revealed species-dependent trends in mechanical performance, with statistically significant differences observed only for tensile strength. Flexural and compressive strengths showed no significant differences among fungal species under the conditions investigated. One-way ANOVA results are summarised in Table 4.

#### 3.6.1. Flexural Strength

One-way ANOVA indicated no statistically significant difference in flexural strength between fungal species (*p* = 0.14). Although *Amauroderma* and *Pycnoporus* exhibited higher mean flexural strength values compared to *Trametes hirsuta*, the observed differences were not statistically significant according to Tukey’s HSD test. The relatively large within-group variance, particularly for the *Amauroderma*-based composites, likely contributed to the absence of statistically significant differences. These results suggest that flexural performance is less sensitive to fungal species selection under the current processing conditions.

#### 3.6.2. Tensile Strength

In contrast, tensile strength was significantly influenced by fungal species (*p* = 0.017). Post hoc Tukey analysis identified a statistically significant difference between *Amauroderma*- and *Trametes hirsuta*-based composites, with *Amauroderma* exhibiting superior tensile strength. No other pairwise comparisons were statistically significant. This finding indicates that fungal species selection plays a critical role in governing tensile load transfer, potentially due to differences in hyphal network morphology, interfacial bonding, or substrate colonisation efficiency.

#### 3.6.3. Compressive Strength

No statistically significant differences were detected in compressive strength among fungal species (*p* = 0.64). Despite *Pycnoporus* exhibiting the highest mean compressive strength, large within-group variability and overlapping confidence intervals resulted in non-significant pairwise comparisons. These results suggest that compressive behaviour is primarily governed by bulk density and panel architecture rather than fungal species alone.

Overall, the statistical outcomes highlight tensile strength as the most species-sensitive mechanical property, while flexural and compressive performance appear more strongly influenced by processing-induced variability and material heterogeneity.

Given the inherent variability of biologically grown composites, increasing the number of replicates beyond the standard minimum (three replicates per test) would improve statistical power and strengthen confidence in the observed trends.

## 4. Conclusions

The research findings provide a comprehensive experimental assessment of MBC production and the mechanical, physical, and chemical properties of panels manufactured using novel fungus–substrate combinations. This study establishes a complete and reproducible methodology for the fabrication and evaluation of MBC panels by systematically investigating the key factors required to produce composites from four locally collected fungal species: *Amauroderma*, *Ganoderma*, *Trametes hirsuta*, and *Pycnoporus*. Rice husks were utilised as the substrate for MBC production for the first time, to the author’s knowledge, demonstrating their suitability as a sustainable, low-cost raw material. By using locally sourced strains, a well-defined rice-husk–wheat nutrient system, and widely accessible hot-pressing conditions, this study provides a reproducible and scalable manufacturing model for MBC production, promoting regional circular economic practices.

By comparing panels produced from different fungal species under identical processing conditions, this study highlights the critical influence of fungal type on composite properties and performance. Thermal analyses confirmed the potential for improved passive fire performance via enhanced char formation of these materials and their suitability as economical and sustainable thermal insulators under the tested conditions. Mechanical testing showed that across species, tensile strength ranged from 0.16 to 0.52 MPa, compressive strength from 0.13 to 0.38 MPa, and flexural strength from 0.60 to 1.38 MPa, with species-dependent differences in stress–strain behaviour. The *Amauroderma*-based composite, derived from a native Queensland species and investigated here for the first time, exhibited the highest Young’s modulus (294 MPa) and the highest tensile strength (0.52 MPa) among all samples. Composites produced from *Amauroderma* and *Pycnoporus* also displayed a more continuous and smoother surface layer, indicating improved cohesion and structural homogeneity compared with the other fungal species.

Furthermore, water absorption tests revealed that panels produced from all four species exhibited low moisture sensitivity and increased surface water repellence relative to previously reported mycelium-derived materials. Analysis of FTIR peak intensity ratios showed higher values for the *Ganoderma*-based composites, suggesting more extensive substrate degradation compared with the other fungal species. In addition, biological stability was assessed by placing all heat-pressed MBC panels produced from the five fungal species on PDA plates under sealed and unsealed conditions and incubating them in darkness at controlled relative humidity for 20 months. No fungal regrowth was observed over the test period, indicating that the heat-pressed composites remained biologically stable with respect to fungal reactivation under the conditions assessed. Statistical analysis confirmed that fungal species selection significantly influenced tensile performance, with *Amauroderma*-based composites exhibiting superior tensile strength (0.52 MPa) compared with *Trametes hirsuta* (0.16 MPa), while no statistically significant differences were observed in flexural or compressive strength based on the statistical criteria applied in this study. Overall, these results indicate that fungal species selection, combined with agricultural waste substrates, can be used to tailor the performance of MBCs for sustainable construction applications. Future work should focus on environmentally friendly strategies to further enhance the mechanical performance of MBCs, enabling their development as reliable alternatives for non-load-bearing construction materials.

## Figures and Tables

**Table 1:** Overview of properties of MBCs made from different fungal species. (±) refers to standard deviation.

Species	Thickness (mm)	Tensile Strength (MPa)	Young’s Modulus (MPa)	Compression Strength (MPa)	Elastic Modulus (MPa)	Flexural Strength (MPa)	Flexural Modulus (MPa)
*Amauroderma* sp.	6.33 ± 1.75	0.52 ± 0.12	294.75 ± 184.42	0.35 ± 0.44	11.46 ± 22.30	0.69 ± 0.39	270.21
*Ganoderma* sp.	5.88 ± 0.25	0.27 ± 0.18	28.66 ± 12.01	0.21 ± 0.15	10.84 ± 7.04	1.19 ± 0.14	352.84
*Pycnoporus coccineus*	5.77 ± 0.26	0.45 ± 0.02	102.14 ± 15.87	0.38 ± 0.19	22.45 ± 13.19	1.38 ± 0.11	532.91
*Trametes hirsuta*	5.45 ± 1.26	0.16 ± 0.07	17.84 ± 59.77	0.13 ± 0.16	6.09 ± 9.65	0.60 ± 0.20	172.95

**Table 2:** Average density values, sample weight and volume of the MBCs produced with different fungal species, substrates and nutrients.

Sample	Average Density (kg/m^3^)	Sample Weight (g)	Sample Volume (cm^3^)
*Pycnoporus coccineus*	250.137 ± 0.0005	0.5097	0.3390 ± 0.0001
*Ganoderma* sp.	251.730 ± 0.0010	0.2984	0.1967 ± 0.0001
*Trametes hirsuta*	250.410 ± 0.0010	0.3783	0.2515 ± 0.0002
*Amauroderma* sp.	259.140 ± 0.0023	0.3133	0.1969 ± 0.0003
*Trametes versicolor*	258.870 ± 0.0005	0.3484	0.2193 ± 0.0001
Wheat grain	245.290 ± 0.0001	1.4529	1.1000 ± 0.0001
Rice husk	235.153 ± 0.0020	0.2184	0.1611 ± 0.0002

**Table 3:** Water contact angle values and wettability status of oven-dried biomass and heat-pressed MBCs.

Fungal Species	Biomass Contact Angle (°)	Wettability Status	Heat-Pressed MBC Contact Angle (°)	Wettability Status
*Amauroderma* sp.	107.2	Hydrophobic (>90°)	140.6	Hydrophobic (>90°)
*Ganoderma* sp.	115.7	Hydrophobic (>90°)	138.3	Hydrophobic (>90°)
*Trametes versicolor*	84.9	More wettable (<90°)	81.3	More wettable (<90°)
*Trametes hirsuta*	114.6	Hydrophobic (>90°)	132.2	Hydrophobic (>90°)
*Pycnoporus coccineus*	112.4	Hydrophobic (>90°)	134.5	Hydrophobic (>90°)

**Table 4:** One-way ANOVA results for mechanical properties.

Property	F-Value	*p*-Value	Significant (α = 0.05)
Flexural	2.40	0.14	No
Tensile	6.28	0.017	Yes
Compression	0.58	0.64	No

## Data Availability

The original contributions presented in this study are included in the article. Further inquiries can be directed to the corresponding author.

## Abbreviations

The following abbreviations are used in this manuscript:

ANOVA	Analysis of Variance
EPS	Expanded Polystyrene
FTIR	Fourier Transform Infrared
GWP	Global Warming Potential
HSD	Honestly Significant Difference
MBC	Mycelium-Based Composite
MOE	Modulus of Elasticity
MOR	Modulus of Rupture
PDA	Potato Dextrose Agar
SEM	Scanning Electron Microscopy
TGA	Thermogravimetric Analysis
WCA	Water Contact Angle
